# Etiology of Influenza-Like Illnesses from Sentinel Network Practitioners in Réunion Island, 2011-2012

**DOI:** 10.1371/journal.pone.0163377

**Published:** 2016-09-21

**Authors:** Elise Brottet, Marie-Christine Jaffar-Bandjee, Ghislaine Li-Pat-Yuen, Laurent Filleul

**Affiliations:** 1 Santé publique France, French national public health agency, Regional unit (Cire) Océan Indien, Réunion, France; 2 Laboratory of virology, Centre Hospitalier Universitaire, Saint-Denis, Réunion, France; Kliniken der Stadt Köln gGmbH, GERMANY

## Abstract

In Réunion Island, despite an influenza surveillance established since 1996 by the sentinel general practitioner’s network, little is known about the etiology of Influenza like-illness (ILI) that differs from influenza viruses in a tropical area. We set up a retrospective study using nasal swabs collected by sentinel GPs from ILI patients in 2011 and 2012. A total of 250 swabs were randomly selected and analyzed by multiplex reverse transcriptase polymerase chain reaction (RT-PCR) including research of 18 viruses and 4 bacteria. We detected respiratory viruses in 169/222 (76.1%) samples, mostly rhinovirus (23.4%), influenza A virus (21.2%), influenza B virus (12.6%), coronavirus (4.9%) and Human metapneumovirus (3.6%). Nine swabs (5.3% of positive swabs) revealed co-infections with two viruses identified, among which six concerned co-infections with influenza viruses. We observed important seasonal differences, with circulation of Human Metapneumoviruses, RSV A and B and coronavirus only during summer; whereas parainfluenza viruses were identified only during winter. In conclusion, this study highlights a substantial circulation of multiple respiratory pathogens in Réunion Island throughout the year. It shows that ILI are not only attributable to influenza and underlines the need for biological surveillance. As the use of multiplex RT-PCR showed its efficacy, it is now used routinely in the surveillance of ILI.

## Introduction

Influenza like-illness (ILI) or acute respiratory infections can be caused by several types of respiratory viruses or bacteria in humans [1]. Influenza viruses, Respiratory Syncytial viruses (RSV) and Parainfluenza viruses are identified as major viruses mostly responsible for ILI and pneumonia in several studies [2]. However practitioners cannot diagnose the infection without a biological test confirmation. Unfortunately, these infections causes are identified in less than 50% [3].

Réunion Island, a French overseas territory with 850,000 inhabitants, is located in the southern hemisphere between Madagascar and Mauritius in the Indian Ocean (Latitude: 21°05.2920 S Longitude: 55°36.4380 E.). The island benefits from a healthcare system similar to mainland France and epidemiological surveillance has been developed by the regional office of the French Institute for Public Health Surveillance (Cire OI), based on the surveillance system of mainland France [4]. Influenza activity generally increases during austral winter, corresponding to summer in Europe [5]. Since 2011, influenza vaccination campaign in Reunion Island starts in April and the vaccine used corresponds to World Health Organization recommendations for the southern hemisphere.

Since 1996, clinical and biological influenza surveillance has been based on a sentinel practitioner’s network [6]. In 2014, this network was composed of 58 general practitioners (GPs) spread over the island and represented around 7% of all Réunion Island GPs. Nasal swabs are randomly collected all along the year and are tested by RT-PCR for influenza viruses. Among these surveillance samples, 40 to 50% are tested positive for influenza A virus, A(H1N1)pdm09 or B virus by the virological laboratory of the University Hospital Center of Réunion. Thus ILI samples tested negative for influenza are of unknown etiology.

Several biological tools allow identifying respiratory pathogens from nasal swab. In recent years, multiplex reverse transcriptase polymerase chain reaction (RT-PCR) has been developed to identify several viruses simultaneously [7–10]. We therefore used this new method to set up a retrospective study using swabs collected by sentinel GPs from 2011 to 2012.

The main objective of our study was to characterize respiratory pathogens responsible for ILI consultations in sentinel GPs in 2011 and 2012. Secondary objectives were to highlight seasonal trends on respiratory pathogens circulation and to describe occurrence of co-infections, especially during the flu season.

## Methods

### Case definition

ILI was defined as a sudden onset of fever more than 38 degrees Celsius and cough, associated or not with other symptoms such as breathing difficulty, headache, etc. Every week, all GPs of the sentinel network were encouraged to collect a nasal swab from the first two patients who presented ILI since less than three days. After being tested for influenza viruses, the 994 swabs collected in 2011 and 2012 are frozen at -80°C at the university hospital center (CHU) laboratory.

### Sampling

Based on the budget, a season-stratified sample of 250 swabs was randomly selected in order to describe circulating viruses including outside flu season. Random sampling was performed with Excel^®^ using the anonymized surveillance database of the Cire OI. The sampling frame contained identification number of swab assigned by Cire OI, laboratory identification number, sex, age, date of onset of symptoms, date of swab collection and result of influenza RT-PCR.

### Virological analyses

We used Respifinder^®^ Smart 22 kits a multiplex RT-PCR (PathoFinder, Maastricht, The Netherlands) which can detect 22 respiratory pathogens. This assay is based on the multiplex ligation-dependent probe amplification (MLPA) technology. The reverse transcription and preamplification steps were performed on the epgradient Mastercycler^®^ (Eppendorf) and the hybridization, ligation and detection steps on the LightCycler^®^480 system (Roche Applied Science). This method was chosen because of its high specificity, compared to other same methods (78% versus 33%) [3,11]. Multiplex analysis allows for rapid production of diagnostic results. It thus allows highlighted the possible presence of eighteen respiratory viruses and four bacteria in one reaction by melt curve analysis: Influenza A not (H1N1)pdm09, Influenza B, Influenza A(H1N1)pdm09v, Respiratory Syncytial Virus A, Respiratory Syncytial Virus B, Parainfluenza 1, Parainfluenza 2, Parainfluenza 3, Parainfluenza 4, Coronavirus OC43, Coronavirus 229 E, Coronavirus NL63, Coronavirus HKU1, Rhinovirus/Enterovirus, Adenovirus, Metapneumovirus humain, Bocavirus, *Chlamydophila pneumoniae*, *Mycoplasma pneumoniae*, *Legionella pneumophila*, *Bordetella pertussis*.

### Statistical methods

Statistical analyses were performed with Stata^®^ and Excel^®^. Two seasons were defined to identify possible seasonal trends in circulation of the viruses: winter season during weeks 23 to 39 between June and September and summer season during the rest of the year.

### Ethics statement

Data and swabs result from a surveillance system that received regulatory approvals, including the CNIL (National Commission for Information Technology and Civil Liberties Number 1592205) approval in July 2012. All the patients have received oral information and gave their consent for swab and data collection. Data were collected for surveillance purpose and are totally anonymous.

## Results

Among the 250 randomly-selected swabs, 26 were not available anymore as they were sent to Influenza Reference Center for confirmation and characterization of the pathogenic agent. According to the sensitivity of the assay two samples could be discordant results between Influenza PCR initially realized and Multiplex PCR. Thus they were deleted from the analysis: one is positive for Influenza in singleplex and negative for all tested pathogens in multiplex and one is positive for Influenza in singleplex and positive for PIV2 in multiplex. In total, 222 analyses were considered. Moreover, 53 samples were negative for all analyzed respiratory pathogens (23.9%) and 169 samples had at least one detected pathogen (76.1%), finally a total of 178 pathogens was identified.

During the study period, a minority of the weeks (21 i.e. 20%) did not include any sampled swab, mainly outside flu season.

Patients’ sex-ratio was 0.63 (86 men and 136 women) and mean age was 28.4 years [min 0; max 81]. Ten percent had less than 5 years, 24% 5–15 years, 63% 15–65 years and only 3% were 65 and older.

The respiratory pathogens most frequently identified in ILI swabs were rhinovirus (23.4%), influenza A not H1N1 (21.2%) and influenza B (12.6%) (Table 1).

**Table 1:** Identified pathogens in nasal swabs analyzed by multiplex PCR, Réunion Island, 2011 and 2012.

	Number of identified pathogens
Rhinovirus/ Enterovirus	52
Influenza A not H1N1	47
Influenza B	28
Coronavirus	11
*OC43*	*4*
*C229E*	*3*
*NL63*	*3*
*HKU1*	*1*
Human metapneumovirus	8
Parainfluenza	7
*PIV1*	*4*
*PIV2*	*1*
*PIV3*	*0*
*PIV4*	*2*
Influenza A(H1N1)pdm09	6
RSV	6
*RSV A*	*4*
*RSV B*	*2*
Adenovirus	5
Mycoplasma pneumoniae	4
Bocavirus	3
Chlamydia pneumoniae	1
TOTAL	178

Among the 22 respiratory pathogens tested by the multiplex, only three were not found in any analyzed sample: Parainfluenza3, *Legionella pneumophila* and *Bordetella pertussis*.

Regarding co-infections, nine swabs revealed the presence of two viruses, among which6 involved influenza viruses (Table 2).

**Table 2:** Identified co-infections in nasal swabs analyzed by multiplex PCR, Réunion Island, 2011 and 2012.

	Number of co-infections
Influenza A—Rhinovirus/Enterovirus	3
Influenza A- Adenovirus	1
Influenza A-Coronavirus 229E	1
Influenza B- Coronavirus 229E	1
Adenovirus- Rhino/Enterovirus	1
Adenovirus-Bocavirus	1
Coronavirus OC 43- Bocavirus	1
TOTAL	9

Analyses showed that some viruses are possibly seasonal and were circulating during a specific period of the year. They are detected only in summer for Human Metapneumovirus, RSV A and B, and influenza A(H1N1)pdm09. For the latter, it is specific to the studied period since the influenza A(H1N1)pdm09 virus reappeared in Réunion Island in October 2012 and was no longer circulating since late 2010. On the opposite, Parainfluenza 1,2 and 4 viruses were identified only in winter. For other pathogens, no specific period of detection was observed.

**Fig 1:**
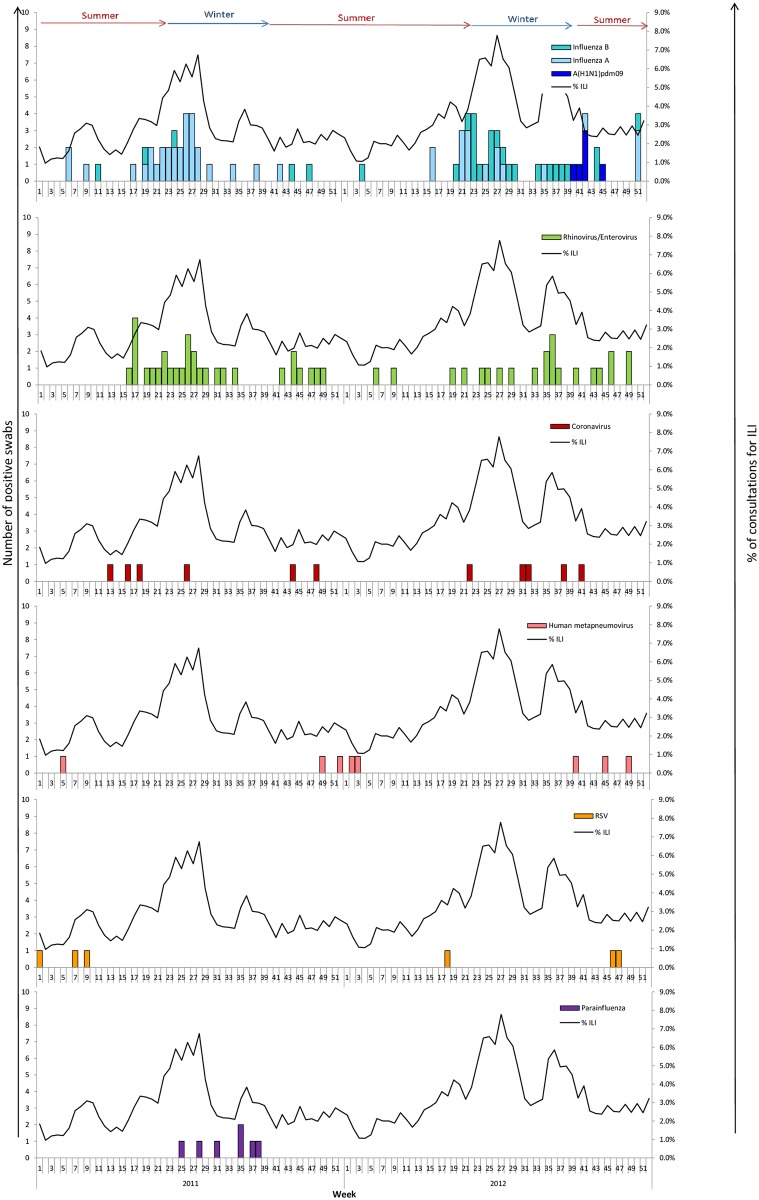
Identified viruses and percentage of ILI consultations declared by the sentinel GPs in 2011 and 2012 in Réunion Island. Specimens from outpatient (n  =  222) that consult for ILI were analyzed using multiplex RT-PCR. Each panel shows the weekly incidence of one virus. For each virus, bars represent the number of specimen detected. The curve showing the weekly proportion of ILI among total visit based on data collected from sentinel network practitioners in 2011–2012 was added to each panel.

A weekly description of samples was realized to study the distribution of respiratory pathogens in 2011 and 2012 (Fig 1). Results of biological analyses were compared with data of ILI consultations declared by sentinel GPs in 2011 and 2012. We observed in 2011, after a first wave in June mainly due to influenza A not H1N1 virus, a second wave of ILI consultations with mainly identification of Parainfluenza viruses and not influenza viruses. In 2012, the second epidemic wave at the end of austral winter coincided with Influenza viruses and Rhinovirus circulation.

**Fig 2:**
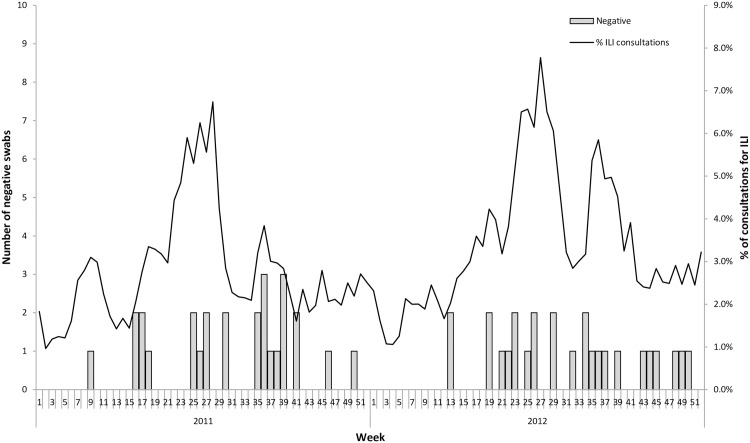
Number of negative swabs and percentage of ILI consultations declared by the sentinel GPs in 2011 and 2012 in Réunion Island. Specimens from outpatient (n  =  222) that consult for ILI were analyzed using multiplex RT-PCR. Bars represent the number of specimen that tested negative for all pathogens analyzed. The curve showing the weekly proportion of ILI among total visit based on data collected from sentinel network practitioners in 2011–2012 was added.

Regarding negative swabs (Fig 2), we observed no seasonality during the study period with a similar proportion whatever the season.

## Discussion

This retrospective study based on a sentinel GPs network showed that not only influenza viruses are responsible for ILI consultations. Indeed, an important circulation of multiple pathogens was observed throughout the year, with 12 different types of pathogens identified in 2011 and 2012. Respiratory viral pathogens were present in 76.1% of samples, which is largely above results from annual influenza surveillance [12]. After influenza viruses, Rhinovirus and Coronavirus were the most common respiratory viruses in Réunion Island.

Although samples were not taken every week, sample was representative of ILI activity and consistent with flu season. Nevertheless, according to the low number of samples, it is difficult to conclude about seasonality. However in our study, RSV was circulating in summer season which is hot and rainy, which is confirmed by other studies in tropical region [13].

This study also highlighted several co-infections, showing that concomitant the multiple etiology of ILI. Co-circulation was already observed in Réunion Island during the A(H1N1)pdm09 pandemic in addition to influenza virus, with identification of other respiratory viruses such as Rhinovirus or Coronavirus [14]. In mainland France, during this pandemic, circulation of major respiratory viruses was found, such as Rhinovirus, Parainfluenza, Coronavirus, Human Metapneumovirus, like in our publication [15–16]. In our study, only 5.3% of positive swabs were co-infections whereas in two studies in Madagascar co-infections represented 27.3% and 29.4% [17–18].

Despite the distance of 9,300 km between Réunion and France, the island is directly connected to Europe with four daily flights to France. These exchanges can impact respiratory pathogens circulation in southern and northern hemisphere. Results of this study can therefore be of interest to both Indian Ocean and Europe countries.

Among the 148 swabs initially negative for influenza because not previously tested for any other viruses, the study found an etiology for 95 swabs. In total, only 53 swabs, representing 24% of the sample, remained without etiology with negative multiplex PCR results all along the year. Multiple hypotheses can explain this result: a poor quality of swabs, preventing from identifying a pathogen, noninfectious causes or other pathogens not included in the multiplex PCR. However, we couldn’t test the negative swabs for RNAse P, a marker of human cells, which could provide a modicum of assurance that the swab contained human cells.

Concerning the two samples divergent for influenza identification between the multiplex and singleplex PCR, we discarded them for the analysis; one was positive in Influenza with singleplex and positive in PIV with multiplex. It could be a false positive result from singleplex. Indeed, as the multiplex PCR assay has a good sensitivity and is considered as a gold-standard, we decided to keep seven negative results for Influenza in singleplex and positive in Influenza in multiplex [7–10].

No case of *Bordetella pertussis* which causes whooping cough and *Legionella pneumophila* which causes Legionnaires’ disease was identified in this study. However, these diseases are rare in Réunion Island, around three cases of Legionnaires’ disease are declared each year.

A limit of the study is that no clinical data were available in the virological surveillance system of influenza in Réunion Island. It was impossible to compare clinical symptoms according to each pathogen and to know if there are different pathogens which cause for instance rhinitis, laryngitis or bronchitis (diseases included in ILI). A specific prospective study including clinical data might provide useful elements in the semiotics of diseases.

In conclusion, this study highlighted an important circulation of multiple pathogens in Réunion Island throughout the year. It shows that ILI is not specific to influenza and so it is essential to have biological results in order to establish the differential diagnosis and thus explain the etiology of symptoms. For a better understanding of respiratory pathogens circulating in Réunion Island, information from this study may also be useful to practitioners who see many patients in consultation with ILI. As the use of multiplex RT-PCR showed its efficacy in the ILI surveillance and allowed to highlight the circulation of other viruses and bacterial causes of respiratory infections, it is now used routinely in the surveillance of ILI. Moreover, it would be interesting to repeat this study every 3 or 5 years adding clinical data to monitor the evolution of respiratory pathogens in Réunion Island over time.

## Supporting Information

S1 File Dataset of identified pathogens per week and percentage of ILI consultations declared by the sentinel GPs in 2011 and 2012 in Réunion Island. (XLSX)
